# Effects of Hydroxypropyl Methylcellulose on Physicochemical Properties and Microstructure of κ-Carrageenan Film

**DOI:** 10.3390/foods11193023

**Published:** 2022-09-29

**Authors:** Jintao Guo, Shuting Dong, Mengyu Ye, Xuan Wu, Xin Lv, Huaide Xu, Mei Li

**Affiliations:** Laboratory of Fruit & Vegetable Storage and Processing, College of Food Science and Engineering, Northwest A&F University, Yangling 712100, China

**Keywords:** κ-carrageenan film, HPMC, physicochemical properties

## Abstract

We investigated the effects of different proportions of hydroxypropyl methylcellulose (HPMC) on the properties of κ-carrageenan film. Biodegradable κ-carrageenan/HPMC films (κCHM film) were prepared by the solution casting method and their physicochemical properties were evaluated. The results show that the addition of HPMC enhanced oxygen barrier capacity, mechanical properties (tensile strength and elongation at break) and thermal stability. Notably, when the addition of HPMC increased to 6% of κ-carrageenan (*w*:*w*), the κCHM-6 film not only effectively improved water resistance, including lower water solubility, water vapor permeability and higher water contact angle, but also made the structure of the κCHM-6 film more compact. Moreover, rheological measurement and atomic force microscopy characterization showed that κ-carrageenan had suitable compatibility with HPMC. Attenuated total reflection–Fourier transform infrared spectroscopy analysis further confirmed the enhancement of hydrogen bond interactions. This finding could contribute to promoting the potential application of κCHM film in food packaging.

## 1. Introduction

Food packaging films play a crucial role in protecting food from physical, chemical and biological hazards. However, the packaging films currently used in the markets are mostly made of petroleum-based polymers such as polyethylene terephthalate (PET), polyvinylchloride (PVC), polyethylene (PE) and polypropylene (PP) [1]. Although these traditional plastic packaging films are popular due to their low cost and convenient operation, the accompanying shortcomings such as non-degradability have caused significant concerns [2]. Moreover, plastic packaging films not only release harmful substances under heating conditions, but also enter the food chain in the form of microplastics and thus endanger human health. Therefore, it is necessary to use safe and biodegradable materials to prepare food packaging films instead of chemically synthesized films [3]. Generally, transparency, barrier properties and mechanical strength are essential parameters in the development of novel food packaging films.

To date, materials obtained from natural renewable sources such as proteins, lipids and polysaccharides have been used to develop biodegradable food packaging films as single or composite film formation. Among these biopolymers, carrageenan is a water-soluble polysaccharide extracted from the cell walls of various marine red algae. It is a linear sulfated polysaccharide composed of alternating units of D-galactose and 3,6-anhydrogalactose connected by α-1,3- and β-1,4-glycosidic linkages. According to the number and position of sulfate groups, carrageenan is mainly classified as κ-carrageenan, ι-carrageenan and λ-carrageenan [4]. Compared with ι-carrageenan and λ-carrageenan, the high gelling capacity of κ-carrageenan makes it an excellent film-forming material. However, the single κ-carrageenan film possesses high hydrophilicity and high brittleness, which greatly limits its application in food packaging [5]. Currently, the incorporation of plasticizers [6] and blending with other polysaccharides [7], proteins [8] or lipids [9] have been used to improve the properties of κ-carrageenan films, but the effects have not been obvious. 

Hydroxypropyl methylcellulose (HPMC), as a cellulose derivative, is widely used in medicine and food owing to its excellent biocompatibility, degradability and film-forming capabilities. It is a linear polysaccharide of β-(1→4)-linked D-glucopyranosyl units, with methyl (-OCH_3_) and hydroxypropyl groups (-OCH_2_CH(OH)-CH_3_) substituted for hydroxyl groups on the branch. Huang et al. [10] found that polyvinyl alcohol films exhibited better mechanical and hydrophobic properties with the addition of HPMC. Brindle et al. [11] presented that the strength and stiffness of whey protein film were increased with the increased concentration of HPMC. Accordingly, HPMC has the potential to improve hydrophilicity and mechanical properties of films. 

Currently, research related to κ-carrageenan and HPMC is mainly focused on the effects of plasticizer types [12], Prunus maackii extract [13] or juice [14], and cork bark extract [15] on κ-carrageenan/HPMC films. However, to the best of our knowledge, the effects of HPMC on the physicochemical properties of κ-carrageenan/HPMC films and the reasons for the improved properties have not been reported. In the present study, we aimed to investigate the impacts of adding small amounts of HPMC on the properties (rheological, barrier, water resistance, mechanical, optical and thermal properties) of κ-carrageenan film, and to speculate the possible reasons for the performance improvement by X-ray diffraction (XRD) spectra, attenuated total reflection–Fourier transform infrared (ATR-FTIR) spectra and microscopic morphological analysis. 

## 2. Materials and Methods

### 2.1. Materials

κ-carrageenan (CAS:11114-20-8) was obtained from Shanghai Yuanye Bio-Technology Co., Ltd. (Shanghai, China). Glycerol (analytical grade) was purchased from Chengdu Chron Chemicals Co., Ltd. (Chengdu, Sichuan, China). Hydroxypropyl methylcellulose (HPMC, CAS:9004-65-3, molecular weight ~22 kDa, 2% aqueous solution viscosity at 20 °C: 40–60 cP) was purchased from Sigma-Aldrich Trading Co., Ltd. (Shanghai, China).

### 2.2. Preparation of κ-Carrageenan/HPMC Films

κ-carrageenan/HPMC films were prepared using a previously reported method [16], with some modifications. Briefly, 1.00 g of κ-carrageenan was firstly dissolved into 100 mL of deionized water with magnetic stirring for 1 h at 40 °C to complete dissolution. Next, 0.30 g of glycerol was added to the 1% (*w*/*v*) κ-carrageenan solution under stirring at 40 °C for 1 h. Subsequently, different proportions of HPMC (0%, 3%, 6%, 9% and 12% of κ-carrageenan, *w*/*w*) were blended into the above solution and continuously stirred for 3 h at 40 °C. After being degassed, the obtained film-forming solutions (10 mL) were cast into a 60 mm diameter glass petri dish, and were dried at 50 °C for 12 h in an oven. The prepared films were labeled as κCHM-0, κCHM-3, κCHM-6, κCHM-9 and κCHM-12 film separately according to the additive proportion of HPMC. All films were equilibrated at 25 °C and 50% relative humidity (RH) for at least 48 h before characterization.

### 2.3. Rheological Measurement of Film-Forming Solutions 

The rheological behavior of the film-forming solution was evaluated using a Discovery HR-1 rheometer (DHR-1, TA Instruments, New Castle, DE, USA) equipped with a flat plate (diameter, 40 mm). The viscoelastic behavior was recorded with the angular frequency ranging from 0.1 to 100 rad/s at a fixed strain of 0.1% under 25 °C. The relationship between the viscosity and the shear rate was studied by increasing the shear rate from 0.01 to 100 s^−1^ at a constant temperature of 25 °C [12,17]. 

### 2.4. Water Resistance

Moisture content (MC) was measured by the gravimetric method with slight modifications [18]. Film samples (20 mm × 20 mm) were weighed (M_1_) and then dried in an oven at 105 °C to a constant weigh (M_2_) before weighing the second time. MC (%) was calculated using Equation (1):

(1) $$\mathrm{MC}\left(\%\right)=100\times \left(M_{1}-M_{2}\right)/M_{1}$$

Water solubility (WS) of films was measured using a previously reported method [19]. Film samples (20 mm × 20 mm) were weighed to determine the initial weight (M_1_), then immersed in 10 mL of distilled water at 25 °C for 12 h. Next, the non-solubilized fractions were dried at 50 °C for 12 h (M_2_), and the water solubility (%) was calculated using Equation (2):

(2) $$\mathrm{WS}\left(\%\right)=100\times \left(M_{1}-M_{2}\right)/M_{1}$$

Water contact angle (WCA) was evaluated with a WCA goniometer (JY-PHb model, Chengde Jinhe Instrument Co., Ltd., Chengde, Hebei, China). First, 10 μL of deionized water released from the automatic precision syringe was dropped on the film sample (20 mm × 20 mm), and the tangent angle between the water drop and the surface of the film was measured after the drop had settled for 5 s on the film [20]. 

### 2.5. Barrier Capacities

Water vapor permeability (WVP) of films was determined following the cup method reported in a previous study [19]. In brief, a glass cup (20 mm in diameter, 45 mm in depth) containing 10 mL of deionized water was sealed by the film samples (30 mm × 30 mm). The area of the film covering the mouth of the cup was 0.000314 m^2^. All the test cups were put into a controlled incubator (25 °C and 50% relative humidity) and weighed every 6 h for 48 h, expressed as a function of time. The partial pressure difference across the films was 1583.84 Pa. WVP was calculated as follows:

(3) $$\mathrm{WVP}\left({g\;\mathrm{Pa}}^{-1}{\;s}^{-1}{\;m}^{-1}\right)=\left(\Delta m\times L\right)/\left(\Delta t\times A\times \Delta p\right)$$

where Δm/Δt is the slope of the curve of weight as a function of time, L (m) is the thickness of the film, A (m^2^) is the area of the film covering the mouth of the cup and Δp (Pa) is the partial pressure difference across the films.

The oxygen permeability (OP) of films was measured using the differential pressure method with a gas permeability tester at 23 °C and 0% RH (Labthink, Perme VAC-V2, Jinan, China) according to the Chinese National Standard GB/T 1038-2000 [21]. The low-pressure chamber was separated from the high-pressure chamber filled with test gas of about 10^5^ Pa by the film. After the film sample was sealed, the air in the low-pressure chamber was pumped to near zero using a vacuum pump. The amount of gas as a function of time from the high-pressure chamber through the film to the low-pressure chamber can be determined by measuring the pressure increment Δp in the low-pressure chamber.

### 2.6. Optical Properties

The light transmittance of the κCHM films was measured using an ultraviolet–visible spectrophotometer (UV-Vis 2550, Shimadzu, Kyoto, Japan) in the wavelength range of 200 to 800 nm, with air as a reference. The opacity of the film was calculated using the following equation [22]:

(4) $$\mathrm{Opacity}=\left(-{\mathrm{logT}}_{600}\right)/x$$

where T_600_ is the percent transmittance at 600 nm and x (mm) is the film thickness.

Color parameters L* (lightness), a* (+, redness; −, green) and b* (+, yellowness; −, blue) were measured with a colorimeter (CM-5, Konica Minolta, Kyoto, Japan) using a standard whiteboard (L = 96.59, a = −0.13, and b = −0.11) as the background reference. The calculation of the total difference (ΔE) was carried out according to Xiao et al. [23]:

(5) $$\Delta E=\sqrt{{\left(L^{*}-L\right)}^{2}+{\left(a^{*}-a\right)}^{2}+{\left(b^{*}-b\right)}^{2}}$$

### 2.7. Mechanical Properties

Tensile strength (TS) and elongation at break (EAB) of film samples (18 × 40 mm) were obtained on a texture analyzer (TA. XT Plus, Stable Micro Systems, London, U.K.) in the tensile mode with an original clamping distance of 20 mm and a stretching rate of 1.0 mm/s [24]. TS (MPa) and EAB (%) were automatically calculated by inputting the width and thickness of the cross-section of film samples through the program set by the device using Equations (6) and (7), respectively. The thickness of the films was measured by a digital micrometer (Meinaite Tools, Shanghai, China) with a precision of 1 μm at ten random locations of the tested film samples [25].

(6) TS=F/A

where F (N) represents the maximum force applied to film sample and A (m^2^) represents the initial cross-sectional area of the tested film.

(7) EAB=ΔL/L

where ΔL (m) is the increase in distance at break and L (m) is the original length between grips of tested film.

### 2.8. Thermogravimetric Analysis (TGA)

Thermal property was analyzed using a thermogravimetric analyzer (TGA 5500, TA Company, New Castle, DE, USA). Film samples were heated from 30 °C to 600 °C at a rate of 20 °C/min under a nitrogen atmosphere. The derivative thermogravimetric analysis (DTG) curves were obtained from the first-order derivative of curves [12].

### 2.9. X-ray Diffraction (XRD) Analysis

The XRD patterns of films were obtained using an X-ray diffractometer (D8 ADVANCE A25, Bruker, Karlsruhe, Germany) with a diffraction angle (2θ) between 10° and 70° at a scanning speed of 5° min^−1^ [26]. 

### 2.10. ATR-FTIR Spectroscopy

The ATR-FTIR spectra of films were recorded by a spectrometer (Vertex 70, Bruker, Karlsruhe, Germany) equipped with an ATR accessory. Each spectrum was obtained in the wavenumber range of 4000 to 650 cm^−1^ by accumulating 32 scans with a spectral resolution of 4 cm^−1^ [27]. 

### 2.11. Morphological Characterization

The surface and cross-sectional microstructure of κCHM films were observed by scanning electron microscopy (SEM, S-3400N, Hitachi, Tokyo, Japan) at 5.00 kV acceleration voltage and 2000× magnification. Samples were fixed on the disc with double-sided tape and coated with a gold layer using the sputter coater (MSP-IS, Hitachi, Tokyo, Japan) before observation [24]. 

The surface topography of films was examined using atomic force microscopy (AFM, Multimode-8, Bruker, Madison, WI, USA). Film samples were fixed on mica disks with double-sided tape. AFM photos of the samples were obtained in the tapping mode with a silicon nitride tip. The values of the average roughness (Ra) and root mean square roughness (Rq) of the films were obtained through NanoScope Analysis (version 1.9, Bruker, Madison, WI, USA) [28]. 

### 2.12. Statistical Analysis

All data were statistically analyzed by one-way analysis of variance (ANOVA) with Minitab 18 Statistical Software (version Minitab^®^ 18.1, Minitab Inc., State College, PA, USA) and were expressed as the mean ± standard deviation. The means were compared by Tukey’s test, and the confidence level was *p* < 0.05. All measurements were performed at least three times.

## 3. Results and Discussion

### 3.1. Rheological Properties

**Figure 1:**
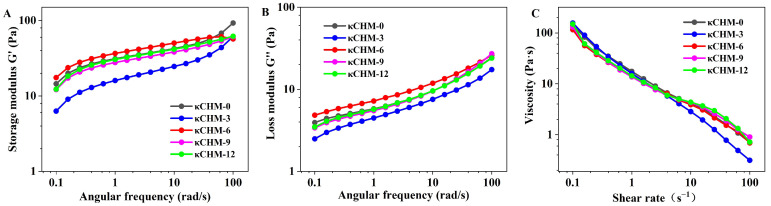
Dynamic rheological properties of κCHM film-forming solutions. (**A**) Storage modulus G′, (**B**) loss modulus G″ and (**C**) viscosity of film-forming solutions.

In general, dynamic rheological properties, including storage modulus G′ (representing elastic behavior) and loss modulus G″ (representing viscous behavior), could reflect the interaction and structural characteristics of various components in the film-forming solutions [29]. In Figure 1A,B, it can be seen that the G′ and G″ of pure κ-carrageenan film-forming solutions increased with the increase in angular frequency, and the G′ value was significantly higher than the G″ value over the entire frequency range. The G′ and G″ still had the same trend after incorporating HPMC, manifesting that all the film-forming solutions were mostly elastic solid-like behavior [30]. The κCHM-6 film-forming solution had the highest G′ and G″ values, indicating that HPMC at a concentration of 6% had a stronger interaction with κ-carrageenan so that the κCHM-6 film had a tighter structure. The κCHM-3 film-forming solution presented the lowest G′ and G″ values, demonstrating that HPMC had fewer interactions with κ-carrageenan. This might lead to a looser film structure compared to other samples.

Figure 1C shows steady shear behavior of κCHM film-forming solutions, that is, the relationship between the change in viscosity and the shear rate. It could be clearly observed that the viscosity of pure κ-carrageenan film-forming solution decreased continuously with the increase in the shear rate, indicating that pure κ-carrageenan film-forming solution exhibited a shear thinning property and was considered to be a non-Newtonian fluid [31]. the film-forming solutions were still non-Newtonian fluids after adding HPMC. At low shear rates, the viscosity of the film-forming solutions containing HPMC was lower than that of the pure κ-carrageenan film-forming solution, which may be explained as the longer side chain of HPMC increasing the molecular free volume and increasing the mobility of the polymer chain [10]. At high shear rates, the entanglement structure among molecules was broken and could not recover for a short time, thus diminishing the viscosity of all film-forming solutions [32]. Notably, the κCHM-3 film-forming solution had the lowest viscosity, further confirming that the interaction between HPMC and κ-carrageenan at 3% concentration was weaker, which was consistent with the results of the G′ and G″ analysis.

### 3.2. Water Resistance

**Figure 2:**
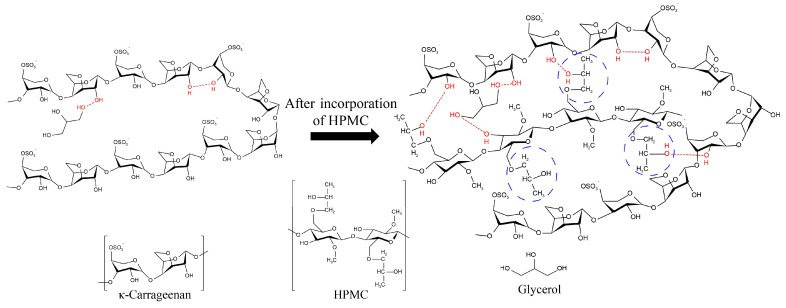
Schematic of possible intermolecular interactions in κCHM films.

Generally, the MC of the film reflects not only the possible influence of the interaction between the components in the film on the affinity of the film to water, but also the ability of each component to hold water molecules [33]. WS of the film provides information about the combination degree among components in composite film [33]. As listed in Table 1, there was no significant difference in MC among all films (*p* > 0.05). WS was obviously decreased with the addition of HPMC (*p* < 0.05). This may be due to the formation of hydrogen bond interactions between HPMC and κ-carrageenan (Figure 2). 

WCA is usually used to evaluate the degree of hydrophobicity of the films [34]. An increase in the water contact angle means a gradual increase in hydrophobicity. The WCA of κCHM films is shown in Table 1. Obviously, the κCHM-6 film had a higher WCA value compared with other samples (*p* < 0.05), indicating that the addition of 6% HPMC significantly improves the hydrophobicity of the pure κ-carrageenan film. This may result from the increased hydrogen bonding between HPMC and κ-carrageenan that reduced the availability of hydrophilic hydroxyl groups on the surface of the film [35], thereby limiting the contact between the film surface and water droplets. Huang et al. [10] also reported the similar effective influence of HPMC on hydrophobic properties of polyvinyl alcohol films by hydrogen bonds between hydroxyl groups of HPMC and polyvinyl alcohol. However, the WCA value decreased when the addition of HPMC exceeded 6%. This may be because some of the hydroxyl groups on the side chain of HPMC were combined with water molecules, which resulted in enhanced hydrophilicity. 

### 3.3. Barrier Properties

WVP is a crucial parameter for the development of novel food packaging films, which shows the ability of water molecules on both sides of the film to permeate the film. It has been reported that WVP of a film is related to relative humidity (RH), type and amount of plasticizer, the integrity of film, hydrophobicity or hydrophilicity, and mobility of polymer chains [36,37]. As shown in Table 1, WVP showed a fluctuant trend with the addition of HPMC when compared to the control group. In particular, WVP of the κCHM-6 film was significantly lower than κCHM-0 film (*p* < 0.05), indicating that WVP of the pure κ-carrageenan film was improved when the addition of HPMC was 6%. This may be because the interaction between HPMC and κ-carrageenan formed a dense and compact structure. Meanwhile, an appropriate amount of HPMC may fill the pores caused by glycerol in the pure κ-carrageenan film structure, further reducing the path of water molecules through the film. Similarly, Roy and Rhim [38] observed that incorporation of mZnONP to κ-carrageenan improved WVP. However, when the HPMC concentration was more than 6%, hydroxyl groups on the side chain of HPMC combined with water molecules, which enhanced the hydrophilicity and led to an increase in WVP.

OP of packaging film is closely related to its effect on food preservation. For a film with high oxygen permeability, too much oxygen could pass through the film and lead to an adverse impact on food [39]. In Table 1, the OP values of films containing HPMC presented an obvious decrease. This might be because the compact and dense film structure allows oxygen to pass through the film in a more complicated and tortuous path. However, as the additive amount of HPMC gradually increased, the OP gradually increased. This may be because excessive HPMC reduced the polarity of films, and thus oxygen as nonpolar molecules could easily penetrate the polarity-reduced film [40]. Overall, the addition of HPMC could improve the oxygen permeability of neat κ-carrageenan film.

### 3.4. Optical Properties

**Figure 3:**
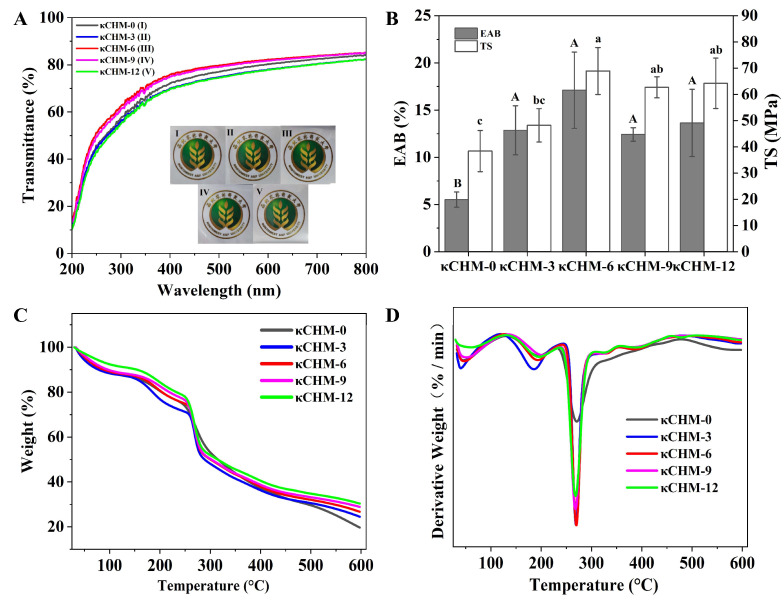
(**A**) Light transmittance, (**B**) mechanical properties, (**C**) TGA thermograms and (**D**) DTG curves of κCHM films. Abbreviations: TS, tensile strength; EAB, elongation at break. The data of TS and EAB are expressed as mean ± standard deviation with *n* = 5. Different letters for the same test parameter indicate significant differences among different groups (*p* < 0.05). TS, tensile strength; EAB, elongation at break.

Light transmittance spectra of films at 200–800 nm is shown in Figure 3A. It can be seen that all films exhibited high transmittance in the visible region. The light transmittance of the film is usually related to the scattering of light, the compatibility of the filler with the matrix and the relative crystallinity of the film [24,33]. As compared to the κCHM-0 film, the κCHM-6 film and the κCHM-9 film showed slightly higher light transmittance. This may be due to the better compatibility between κ-carrageenan and HPMC. Furthermore, the calculated opacity at 600 nm is listed in Table 2. There was no significant difference in the opacity of all films (*p* > 0.05); that is, the addition of HPMC had no effect on the opacity of the film.

Apparent color and transparency of a film play important roles in food packaging. The color parameters of κCHM films are shown in Table 2. In this study, the L*, a*, b* and ΔE values of the films containing HPMC did not change significantly compared with the pure κ-carrageenan film (κCHM-0 film) (*p* > 0.05), indicating that the addition of HPMC does not affect the color of the κ-carrageenan film. At the same time, it showed that HPMC has suitable miscibility or compatibility with κ-carrageenan [41]. Kassab et al. [42] also reported that the transparency level of the pure κ-carrageenan film did not change after the addition of CNC. All composite films were transparent films, which is beneficial for consumers to better observe the food. 

### 3.5. Mechanical Properties

The thicknesses of all κCHM film formulations are shown in Table 1. There was no significant difference among all films, with thickness ranging from 39 to 41 μm (*p* > 0.05). Generally, the TS value represents the maximum tensile stress that the film could sustain and EAB represents the maximum change in the length of the film before breaking. As shown in Figure 3B, the EAB of κCHM-0 film was only 5.51 ± 0.80%, indicating that the pure κ-carrageenan film was very brittle. When adding HPMC, the EAB values of the film exceeded 12%, reflecting that the flexibility of the κ-carrageenan film was significantly improved. This is probably attributed to the hydroxypropoxy groups in the branched chain of HPMC increasing the free volume of chains and facilitating the movement of polymer molecular chains [10]. In addition, the TS values also increased significantly from 38.39 ± 7.84 MPa for the κCHM-0 film to 68.90 ± 8.95 MPa for the κCHM-6 film with the addition of HPMC. This could be explained by the fact that a stronger network structure was formed through intermolecular and intramolecular hydrogen bonds. Moreover, the compact and dense film structure, as well as compatibility between κ-carrageenan and HPMC, were also conducive to the improvement of TS. Similarly, Huang et al. [10] reported that the introduction of HPMC could significantly improve the mechanical properties of polyvinyl alcohol films.

### 3.6. Thermal Stability

The thermal characteristics of κCHM films are depicted in Figure 3C,D. It can be observed that κCHM-0 film had three obvious stages of thermal degradation. The first stage appeared at around 50 °C, representing the evaporation of water within the film matrix [43]. The second stage from 130.55 °C to 229.73 °C was caused by glycerol decomposition, and the third stage from 233.04 °C to 316.81 °C was ascribed to the κ-carrageenan decomposition. Furthermore, for the films containing HPMC, there was another thermal degradation stage between 312.35 °C and 483.83 °C, which was associated with the decomposition of HPMC [14]. After the final thermal degradation, residuals of κCHM-0, κCHM-3, κCHM-6, κCHM-9 and κCHM-12 films were 19.66%, 24.51%, 26.72%, 28.90% and 30.37%, respectively. This demonstrated that the addition of HPMC enhanced the thermal stability of neat κ-carrageenan film [42]. A similar improvement of HPMC with polyvinyl alcohol composite film was reported by Huang et al. [10]. We speculated that the formation of hydrogen bonds between HPMC and κ-carrageenan might make the internal structure more stable, thereby reducing decomposition. Moreover, the minerals in HPMC may contribute to improving the thermal stability of κCHM films after mixing. 

### 3.7. XRD Analysis

**Figure 4:**
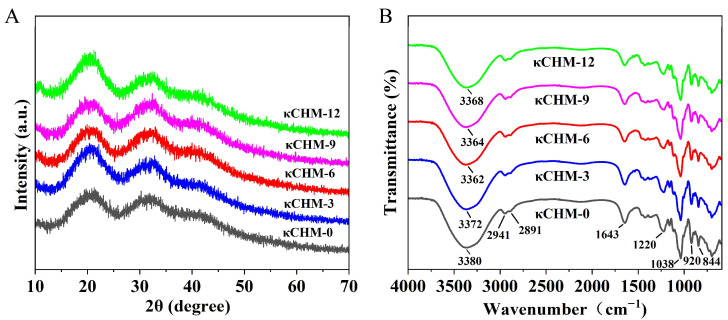
(**A**) XRD pattern and (**B**) ATR-FTIR spectrum of κCHM films. XRD, X-ray diffraction analysis; ATR-FTIR, attenuated total reflection–Fourier transform infrared spectroscopy.

The diffraction pattern of all κCHM films exhibited two clear diffraction peaks around 20.4° and 32.5° (Figure 4A). For the pure κ-carrageenan film, the broad and diffuse peak reflected the amorphous structure of the κ-carrageenan [44]. Its relative crystallinity was 15.29%. The incorporation of HPMC into the κ-carrageenan-based films did not generate new diffraction peaks in the XRD patterns, indicating that HPMC did not considerably change the amorphous structure of the κ-carrageenan. The significant increase in peak intensity of κCHM-3 and κCHM-12 films at diffraction peaks suggested an increase in crystallinity (17.29% for κCHM-3 film, 17.91% for κCHM-12 film). The increase in crystalline aggregates hindered the transmission of light [33]. These data could further explain why the κCHM-3 and κCHM-12 films had lower light transmittance. The changes in peak intensity of κCHM-6 and κCHM-9 films were not clear. The relative crystallinities were 15.51% and 16.02%, respectively. κCHM-6 film had lower relative crystallinity; thus, we suggest that 6% HPMC had better compatibility with κ-carrageenan.

### 3.8. ATR-FTIR

The chemical interaction between κ-carrageenan and HPMC was analyzed by ATR-FTIR. In Figure 4B, the characteristic peak in the range of 3000–3700 cm^−1^ is attributed to the O-H stretching vibration of carrageenan [45]. The weak band at around 2941 cm^−1^ and 2891 cm^−1^ correspond to the C-H stretching vibrations of alkane groups present in the carrageenan chain, while a peak observed at 1643 cm^−1^ is due to stretching vibrations of C=O in the D-galactose in κ-carrageenan [13]. A peak appeared at 1220 cm^−1^, representing the presence of sulfate ester groups in κ-carrageenan [46]. The band at 1038 cm^−1^ is assigned to C-C and C-O stretching vibrations [42]. The prominent peaks at 920 cm^−1^ and 844 cm^−1^ arose from C-O of 3,6-anhydro-D-galactose and C-O-SO_3_ of D-galactose-4-sulfate [47,48]. Compared with the κCHM-0 film, the peak at about 3380 cm^−1^ shifted to a lower wavenumber with the addition of HPMC, demonstrating the enhancement of hydrogen bonds in the film matrix. For the κCHM-6 film, the peak at 3380 cm^−1^ was obviously shifted to 3362 cm^−1^, suggesting that more intermolecular hydrogen bonds had been formed between κ-carrageenan and HPMC. This may be one of the reasons why the κCHM-6 film possessed larger WCA and lower WS and WVP. 

### 3.9. Microstructure

**Figure 5:**
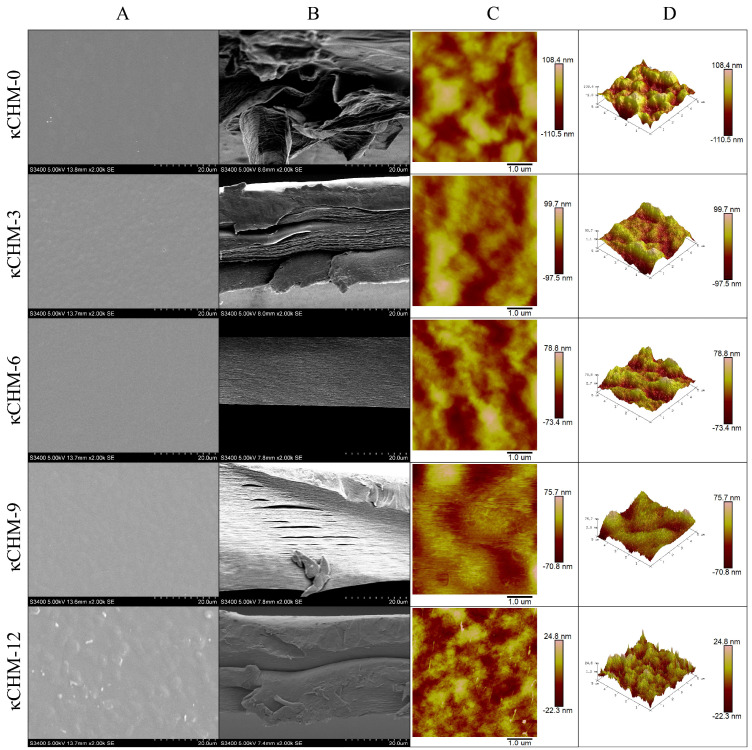
Morphological observations using scanning electron microscopy (SEM) and atomic force microscopy (AFM). (**A**) SEM images of surface and (**B**) cross-section of κCHM films. (**C**) AFM-2D and (**D**) AFM-3D images of the surface morphology of κCHM films.

Figure 5 shows the surface and cross-sectional SEM images and surface topography of κCHM films. As seen in Figure 5A,B, the κCHM-0 film shows a relatively smooth surface, and exhibits discontinuous internal structure with large cavities. The latter may be related to the addition of glycerol, which weakens the interaction between κ-carrageenan molecules, increases the free volume of molecules and forms cavities [32]. With the addition of HPMC, the surface of the film changed significantly. It can be seen that there were visible bulges similar to bubbles on the surface of the κCHM-3 film and the κCHM-12 film, and white aggregations appeared on the surface of the κCHM-12 film (Figure 5B). These bulges may affect the optical properties of the films. As presented in Figure 5C, the κCHM-6 film shows a flat and smooth surface, demonstrating that there was better compatibility between HPMC and κ-carrageenan when the HPMC concentration was 6%. Moreover, the internal structure of the κCHM-6 film was the most compact and dense (Figure 5B). A smoother surface reduced light scattering and internal ordered film structure reduced obstacle of light transmission through the film; this might contribute to higher light transmittance of κCHM-6 and κCHM-9 films [24]. On the contrary, the cross-sectional images of other HPMC-containing film formulations showed varying degrees of discontinuity and even severe delamination. Compared with the κCHM-0 film, the internal cavities of the HPMC-containing films gradually disappeared, which may be because the addition of HPMC filled these pores, cross-linked and reorganized the polymer molecules, and thus changed the film structure.

The surface topography of κCHM films is presented in Figure 5C,D. As illustrated in AFM-2D images (Figure 5C), the dark and light areas can be clearly observed in the whole images, displaying the peaks and valleys of the film surface, respectively [49]. From the AFM-3D images (Figure 5D), it can be directly observed that the surface morphology of the film has changed. As the concentration of HPMC gradually increased, thorn-like bulge structures gradually appeared on the surfaces of the film. κCHM-9 and κCHM-12 films had the most obvious structure. We speculated that the thorn-like bulge structure had a larger surface area in contact with water molecules, which may increase WVP values of the κCHM-9 film and the κCHM-12 film. Meanwhile, Rq and Ra values gradually decreased after the incorporation of HPMC (*p* < 0.05) (Table 3), further confirming the compatibility between κ-carrageenan and HPMC. 

## 4. Conclusions

In this work, κCHM films were prepared by the incorporation of HPMC as reinforcing agents into κ-carrageenan and were characterized. The color, opacity, thickness as well as moisture content of κ-carrageenan films did not change significantly after adding HPMC from 3% to 12%. In particular, the presence of 6% HPMC (*w*/*w*) brought an obvious improvement in the mechanical strength (38.39 to 68.90 MPa for TS, 5.51% to 17.1% for EAB), water contact angle (39.5° to 88.6°) and thermal stability of κ-carrageenan films. Moreover, when adding 6% HPMC (*w/w*), the κCHM-6 film possessed lower water solubility (10.40% to 8.43%), water vapor permeability (8.73 to 7.71 × 10^−11^g·Pa^−1^·s^−1^·m^−1^) and oxygen permeability (0.228 to 0.123 cm^3^/m^2^·24 h·0.1 MPa) and had a smoother film surface and more compact structure. The FTIR results of the κCHM-6 film indicated that more intermolecular hydrogen bonds formed between κ-carrageenan and HPMC. Therefore, we suggest that adding an appropriate amount of HPMC can improve the properties of κ-carrageenan film, and we expect κCHM-6 film to be a new type of biodegradable and environmentally friendly food packaging material.

## Figures and Tables

**Table 1:** Thickness, MC, WS, WCA, WVP and OP of κCHM films.

Samples	Thickness (μm)	MC (%)	WS (%)	WCA (°)	WVP (10^−11^g·Pa^−1^·s^−1^·m^−1^)	OP (cm^3^/m^2^·24 h·0.1·MPa)
κCHM-0	39 ± 4 ^a^	18.56 ± 2.90 ^a^	10.40 ± 1.25 ^a^	39.5 ± 1.9 ^c^	8.73 ± 0.16 ^b^	0.228
κCHM-3	39 ± 4 ^a^	17.23 ± 3.55 ^a^	10.18 ± 0.94 ^ab^	43.4 ± 2.5 ^c^	9.41 ± 0.37 ^a^	0.122
κCHM-6	38 ± 4 ^a^	16.51 ± 2.51 ^a^	8.43 ± 0.99 ^bc^	88.6 ± 2.8 ^a^	7.71 ± 0.12 ^c^	0.123
κCHM-9	38 ± 5 ^a^	17.31 ± 2.37 ^a^	7.62 ± 0.48 ^c^	79.8 ± 8.1 ^ab^	8.48 ± 0.17 ^b^	0.131
κCHM-12	41 ± 4 ^a^	16.44 ± 1.87 ^a^	6.82 ± 0.63 ^c^	69.6 ± 6.7 ^b^	9.52 ± 0.04 ^a^	0.164

Different letters in the same column indicate significant differences (*p* < 0.05). The data are expressed as mean ± standard deviation (*n* = 10 for thickness, *n* = 4 for MC, WS, WCA and WVP). MC, moisture content; WS, water solubility; WCA, water contact angle; WVP, water vapor permeability; OP, oxygen permeability.

**Table 2:** Color parameters and opacity of κCHM films.

Samples	L*	a*	b*	ΔE	Opacity (A/mm)
κCHM-0	97.15 ± 0.11 ^ab^	−0.02 ± 0.01 ^ab^	0.54 ± 0.04 ^b^	0.86 ± 0.08 ^abc^	2.39 ± 0.13 ^a^
κCHM-3	97.36 ± 0.24 ^a^	0.00 ± 0.02 ^a^	0.54 ± 0.12 ^b^	1.04 ± 0.13 ^a^	2.58 ± 0.41 ^a^
κCHM-6	96.93 ± 0.09 ^b^	−0.03 ± 0.01 ^b^	0.59 ± 0.03 ^ab^	0.79 ± 0.01 ^c^	2.27 ± 0.35 ^a^
κCHM-9	96.97 ± 0.12 ^b^	−0.03 ± 0.00 ^b^	0.62 ± 0.04 ^ab^	0.83 ± 0.07 ^bc^	2.39 ± 0.15 ^a^
κCHM-12	97.19 ± 0.08 ^ab^	−0.02 ± 0.02 ^ab^	0.70 ± 0.07 ^a^	1.01 ± 0.10 ^ab^	2.69 ± 0.86 ^a^

Different letters in the same column indicate significant differences (*p* < 0.05). The data are expressed as mean ± standard deviation (*n* = 3).

**Table 3:** Rq and Ra of the surface morphology of κCHM films.

Samples	Rq (nm)	Ra (nm)
κCHM-0	32.33 ± 4.74 ^a^	25.40 ± 3.73 ^a^
κCHM-3	23.67 ± 4.38 ^b^	18.90 ± 3.08 ^b^
κCHM-6	21.20 ± 0.72 ^b^	17.10 ± 0.69 ^b^
κCHM-9	19.97 ± 1.14 ^b^	13.93 ± 0.66 ^b^
κCHM-12	7.78 ± 1.55 ^c^	6.04 ± 1.10 ^c^

Different letters in the same column indicate significant differences (*p* < 0.05). The data are expressed as mean ± standard deviation (*n* = 3). Rq, root mean square roughness; Ra, average roughness.

## Data Availability

The data presented in this study are available on request from the corresponding author.
